# Baseline Apparent Diffusion Coefficient as a Predictor of Response to Liver-Directed Therapies in Hepatocellular Carcinoma

**DOI:** 10.3390/jcm7040083

**Published:** 2018-04-14

**Authors:** Andrew Niekamp, Reham Abdel-Wahab, Joshua Kuban, Bruno C. Odisio, Armeen Mahvash, Manal M. Hassan, Aliya Qayyum, Ahmed Kaseb, Rahul A. Sheth

**Affiliations:** 1 Department of Interventional Radiology, Division of Diagnostic Imaging, MD Anderson Cancer Center, Houston, TX 77030, USA; Andrew.S.Niekamp@uth.tmc.edu (A.N.); jdkuban@mdanderson.org (J.K.); BCOdisio@mdanderson.org (B.C.O.); armeen.mahvash@mdanderson.org; (A.M.); 2 Department of Gastrointestinal Medical Oncology, MD Anderson Cancer Center, Houston, TX 77030, USA; raali@mdanderson.org (R.A.-W.); mhassan@mdanderson.org (M.M.H.); akaseb@mdanderson.org (A.K.); 3 Division of Diagnostic Imaging, MD Anderson Cancer Center, Houston, TX 77030, USA; AQayyum@mdanderson.org

**Keywords:** hepatocellular carcinoma, diffusion weighted imaging, chemoembolization

## Abstract

Predicting outcomes in patients with hepatocellular carcinoma (HCC) who undergo locoregional therapies remains a substantial clinical challenge. The purpose of this study was to investigate pre-procedure diffusion weighted magnetic resonance imaging (DW-MRI) as an imaging biomarker for tumoral response to therapy for patients with HCC undergoing drug eluting embolic (DEE) chemoembolization and radioembolization. A retrospective review of HCC patients who underwent DEE chemoembolization or radioembolization was performed. Of the 58 patients who comprised the study population, 32 underwent DEE chemoembolization and 26 underwent radioembolization. There was no significant difference in median apparent diffusion coefficient (ADC) values across the two treatment groups (1.01 × 10^−3^ mm^2^/s, *P* = 0.25). The immediate objective response (OR) rate was 71% (40/56). Tumors with high ADC values were found to have a higher probability of OR within 90 days (odds ratio 4.4, *P* = 0.03). Moreover, index lesion specific progression free survival (PFS) was greater for high ADC tumors, independent of conventional predictors of treatment response (hazard ratio 0.44, *P* = 0.01). Low ADC was associated with poorer PFS (*P* = 0.02). Pre-procedure ADC < 1.01 × 10^−3^ mm^2^/s is an independent predictor of poorer immediate OR and index lesion specific PFS in patients with HCC undergoing DEE chemoembolization or radioembolization.

## 1. Introduction

Predicting outcomes in patients with hepatocellular carcinoma (HCC) who undergo locoregional therapies remains a substantial clinical challenge. Contemporary prognostic tools include classification systems such as the Barcelona Clinic Liver Cancer (BCLC) system that rely on clinical, serologic, and tumor morphologic data to group patients into broad categories. While these categories correlate with mortality rates and provide a framework upon which to make treatment decisions, they are undermined by the wide range of outcomes that can occur within each category. For example, the defining criteria for intermediate-stage HCC circumscribe a highly heterogeneous group of patients, and as a result, outcomes following standard of care chemoembolization therapy vary widely, with median survival rates ranging from 20–22 months [1,2] to 48 months [3].

There is an unmet need for a noninvasive imaging tool that can provide a priori predictions on tumor treatment response following locoregional therapies. Diffusion weighted magnetic resonance imaging (DW-MRI) is a well-established imaging tool that has been applied towards predicting tumor response following chemoembolization. For example, early changes in apparent diffusion coefficient (ADC) values after chemoembolization have been shown to predict long term outcomes [4,5]. DW-MRI is an appealing imaging tool as it can be readily performed on conventional MRI systems and can report on fundamental tumor biology. Diffusion weighted imaging measures the random Brownian motion of water molecules in both the intracellular and extracellular compartments, the latter of which includes the interstitial and intravascular spaces. As a result, quantitative ADC maps calculated from diffusion weighted images can serve as surrogates for several critical biological properties of tumors. For example, ADC values are inversely correlated with tumor cellular density, as highly dense tumors restrict diffusion. As a result, DW-MRI is a surrogate for tumor interstitial pressure (TIP) [6], a major barrier to cancer therapy [7,8,9]. Thus, DW-MRI has the potential to serve as a non-invasive imaging biomarker for tumor responsiveness to locoregional therapies.

The purpose of this study was to interrogate pre-procedure DW-MRI as an imaging biomarker for tumoral response to therapy for patients with HCC undergoing DEE chemoembolization and radioembolization. Tumoral ADC values were assessed as predictors of immediate overall response (OR) as well as progression free survival (PFS). The relative efficacy of DEE chemoembolization and radioembolization in lesions identified as poor responders based on their ADC values was also evaluated.

## 2. Material and Methods

### 2.1. Study Population

Institutional review board approval was obtained for this single institution retrospective cohort study. Data collection and analysis were performed in compliance with regulations of the Health Insurance Portability and Accountability Act. An institutional radiology database was queried for all patients who underwent DEE chemoembolization and radioembolization for HCC between 2012 and 2015. HCC tumors were diagnosed by imaging criteria defined by the American Association for Study of Liver Disease guidelines or by biopsy. Patients who underwent MR imaging which included DW imaging with calculation of ADC maps within 60 days of the procedure were included. Patients without at least 1 cross-sectional imaging follow up study were excluded. Patients who underwent radiation therapy, DEE chemoembolization, radioembolization, or ablation of the index lesion prior to the index procedure were excluded. Patients with index lesions smaller than 1cm were excluded.

A total of 148 patients were identified who underwent MRI at our institution within 6 months prior to DEE chemoembolization or radioembolization for HCC. After applying the exclusion criteria above, a total of 58 patients (32 DEE chemoembolization, 26 radioembolization) comprised the study population. The demographics for these patients are summarized in Table 1.

### 2.2. MRI and DWI Protocol

Imaging was performed on 1.5-T and 3.0-T scanners (Siemens, Erlangen, Germany). Liver MRI protocol included axial T1-weighted dual echo in-phase and out-of-phase imaging and axial T2 balanced gradient echo (TrueFISP). Dynamic contrast-enhanced gradient-echo T1-weighted images with fat suppression were obtained at 4 timepoints: pre-contrast, arterial phase (8 s after contrast administration), venous phase (70 s after contrast administration), and delayed phase (180 s contrast administration). The intravenous contrast agents used during the study period were Gadavist (Bayer, Berlin, Germany) and Eovist (Bayer). The contrast injection was performed with 10 cc of the contrast agent injected at a rate of 2 cc/sec, followed by a 10 cc saline flush.

DW imaging consisted of fat-suppressed single-shot echo planar sequences obtained during breath holds and performed at b-values of 50, 400, and 800 s/mm^2^. ADC maps were automatically calculated and displayed on a standard picture archiving and communication system (iSite, Philips, Amsterdam, The Netherlands).

### 2.3. Procedural Techniques

The selection of the treatment modality for individual patients was based upon an institutional protocol as well as multidisciplinary conference discussions involving interventional radiologists, oncologists, and surgical oncologists. All procedures were performed by fellowship-trained interventional radiologists with experience ranging from 2 to >15 years.

### 2.4. DEE Chemoembolization

All DEE chemoembolization procedures were performed via a transfemoral approach. The selection of the calibrated microsphere embolic agent was at the operator’s discretion and included 100–300 micron LC Bead microspheres (Biocompatibles/BTG, Farnham, UK) (16/34, 47%), 75–150 micron LC Bead microspheres (16/34, 47%), 40 micron Oncozene microspheres (Boston Scientific, Natick, MA, USA) (1/34, 3%) and 75 micron Oncozene microspheres (1/34, 3%). DEE transarterial chemoembolization was performed after digital subtraction angiography (DSA) of the celiac and superior mesenteric arteries with a 5-Fr catheter. Selective catheterization of higher order branches was performed with a coaxial 2.4 or 2.8-Fr microcatheter (Renegade STC or Renegade Hi-Flo, respectively; Boston Scientific, Natick, MA, USA). DSA was repeated after selective catheterization to ensure tumor blush. Cone-beam CT angiography was performed in all procedures to identify target arterial branches. DEE transarterial chemoembolization was administered as selectively as possible. The endpoint for each procedure was stasis within the supplying arteries. The dose of doxorubicin varied with the volume of embolic agent that was administered. In 5 patients (5/34, 15%), stasis was not achieved following the administration of the prescribed DEE dose, and therefore embolization with calibrated microspheres (Embozene, Boston Scientific, Natick, MA, USA) was performed until stasis was achieved.

### 2.5. Radioembolization

All patients underwent a standardized workup including laboratory and imaging assessment as well as a mapping procedure with technetium-99m macroaggregated albumin, as previously described [10]. Dosimetry and infusion protocols was performed according to manufacturer guidelines using the partition model [11]. All radioembolization procedures were performed via a transfemoral approach. All patients underwent glass based radioembolization with the exception of 1 patient who underwent resin based radioembolization due to elevated liver enzymes. Lobar treatment was performed when segmental feeding vessels were not clearly seen. Following delivery of the treatment dose, patients underwent yttrium-90 Bremsstrahlung single-photon emission computed tomography (SPECT)/CT imaging to measure liver dosimetry and assess for extrahepatic activity.

### 2.6. Image Analysis

Index HCC lesions were selected in the following manner. For patients who underwent DEE chemoembolization, pre-procedural imaging and intra-procedural angiography were reviewed to identify one lesion per patient that was embolized with drug-eluting embolics. For patients who underwent radioembolization, review of the Bremsstrahlung SPECT/CT imaging immediately following the radioembolization procedure was performed to identify an index lesion within the treated area.

ADC values for index lesions were measured by an interventional radiologist with subspecialty training in abdominal imaging and 8 years of experience in liver MRI interpretation. The reader was blinded to the procedural outcome. An automatically generated, pixel-wise ADC map from the pre-procedure MRI was reviewed. As tumors may be difficult to visualize on ADC maps alone, the index lesion were localized on the ADC map using contrast-enhanced sequences. A region of interest circumscribing the entire lesion was drawn, and the mean and standard deviation of the lesion’s ADC value were recorded.

Response to therapy was evaluated on post-procedure triphasic contrast-enhanced CT and MRI scans by two radiologists with combined 10 years of experience in abdominal imaging. These readers were blinded to the pre-procedure ADC values but were aware of the location of the index lesion. Discrepancies were resolved by the two radiologists reviewing the images together and reaching a consensus. Response was evaluated using unidimensional mRECIST criteria on a single lesion basis. OR was defined as either complete response (CR) or partial response (PR). Treatment response was evaluated on the first post-procedure imaging study as well as all subsequent imaging studies. Only patients with cross-sectional imaging within 90 days following the index procedure were included in the evaluation of immediate objective response. PFS was defined as the time interval between the pre-procedure MRI study and the observation of progressive disease (PD) or patient death, whichever occurred first. Of note, treatment response was evaluated only on the index lesion. That is, PD was defined as an increase of at least 20% of viable/enhancing tissue in the index lesion; increase in size of non-index lesions or the development of additional lesions was not considered PD.

### 2.7. Covariates

Patient clinical and imaging characteristics were collected and included age, gender, index lesion size (longest axial measurement on pre-procedure MRI), presence of ascites, single versus multiple tumors, presence of vascular invasion, presence of lymph node metastases, presence of extra-hepatic metastases, extra-hepatic metastases (ECOG) status, history of hepatic encephalopathy, pre-procedure serum total bilirubin, pre-procedure serum albumin, Childs-Pugh score, and BCLC classification.

### 2.8. Statistical Analysis

Univariate analysis of demographic, laboratory, and imaging characteristics of patients who underwent DEE chemoembolization and radioembolization was performed using the Fisher exact test and Wilcoxon rank sum test, as appropriate. Multivariate logistic regression was performed to identify independent predictors for OR in the first post-procedure imaging study. Cox proportional hazards and Kaplan-Meier models were used to evaluate ADC value as an independent predictor of PFS. Covariates found on univariate analysis to have *P* < 0.10 were included the Cox proportional hazard analysis. The log-rank test was used to evaluate the significance of the Cox model. All statistical analyses were performed using the standard statistical software package R (The R Foundation). A cutoff value of *P* < 0.05 was used for statistical significance.

## 3. Results

Of the 58 patients who comprised the study population, 32 underwent DEE chemoembolization, 26 underwent radioembolization (Table 1). The study population was predominantly male (46/58, 79%), with a median age of 63 years. The median dose of doxorubicin delivered during DEE chemoembolization was 40 mg (range 5–100 mg). The median tissue absorbed dose delivered during radioembolization was 104.8 Gy (range 71.2–136.9).

Across the two treatment modality groups, patients who underwent radioembolization had larger lesions compared to those who underwent DEE chemoembolization (*P* < 0.001). DEE chemoembolization patients (*P* = 0.007) had a lower rate of extrahepatic disease compared to patients who underwent radioembolization. Likewise, DEE chemoembolization patients (*P* = 0.005) had a lower rate of lymph node metastases relative to radioembolization patients. The median ADC value for the study population was 1.01 × 10^−3^ mm^2^/s, and there was no significant difference in median ADC values across the two treatment groups (*P* = 0.25).

**Figure 1:**
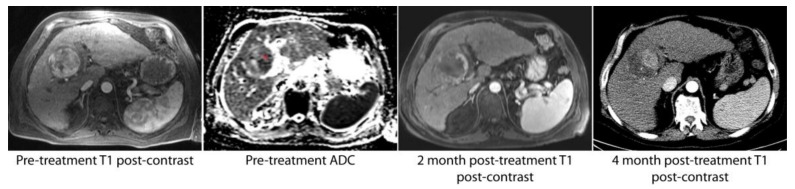
A solitary hepatocellular carcinoma (HCC) lesion with relatively homogenous arterial enhancement was found to have heterogeneous apparent diffusion coefficient (ADC) signal intensity on pre-procedure imaging. In particular, the medial aspect of the lesion was hypointense relative to the lateral aspect (red asterisk). The first post-procedure imaging study at 2 months demonstrated a small area of residual tumor in the portion of the tumor noted to have low ADC values. At 4 months, there was progression of disease in this same location.

The median duration of time between the index procedure and the first available cross-sectional imaging study to assess immediate OR was 55.5 days (range: 17–204 days). A total of 2 patients (2/58) were excluded from the immediate OR analysis due to the lack of imaging within 90 days of the index procedure. For the remaining 56 patients, the immediate OR rate was 71% (40/56). Of numerous demographic and imaging variables, the only statistically significant variable associated with OR on multivariate analysis was the index tumor’s ADC value (Table 2). That is, the probability of OR was higher (odds ratio 4.4, *P* = 0.03) for tumors with high ADC values, thresholded by the median ADC value of 1.01 × 10^−3^ mm^2^/s (Figure 1).

Risk factors for index lesion specific PFS were next evaluated. Based on univariate analysis, presence of extra-hepatic disease (*P* = 0.05), presence of ascites (*P* = 0.05), presence of lymph node metastases (*P* = 0.006), and high ADC value (*P* = 0.03) were all included in the multivariate Cox proportional hazards analysis. Multivariate analysis identified high ADC value (hazard ratio 0.44, *P* = 0.01) to be significantly associated with a lower rate of progression or death occurring compared to a low ADC value (Table 3). The Cox model was found to be statistically significant (*P* = 0.0003).

**Figure 2:**
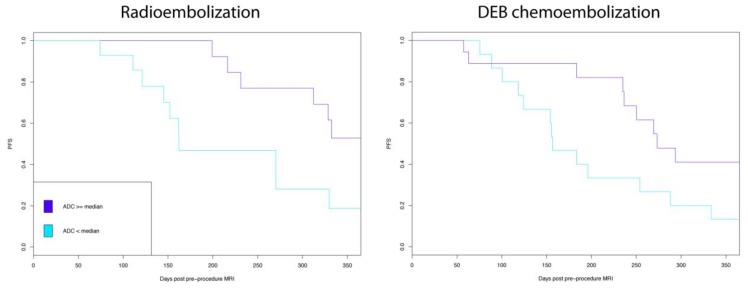
Low apparent diffusion coefficient (ADC) values were significantly associated with poorer index lesion specific progression free survival (PFS) for both radio embolization and drug eluting embolic (DEE) chemoembolization.

The influence of ADC values within treatment modalities on PFS and survival was next assessed. Low ADC was associated with poorer PFS for both the DEE chemoembolization subgroup (*P* = 0.02) and the radioembolization subgroup (*P* = 0.02) (Figure 2). However, low ADC value was not significantly associated with survival for the study population (*P* = 0.95) or for the subgroups of DEE chemoembolization (0.87), or radioembolization (*P* = 0.93).

**Figure 3:**
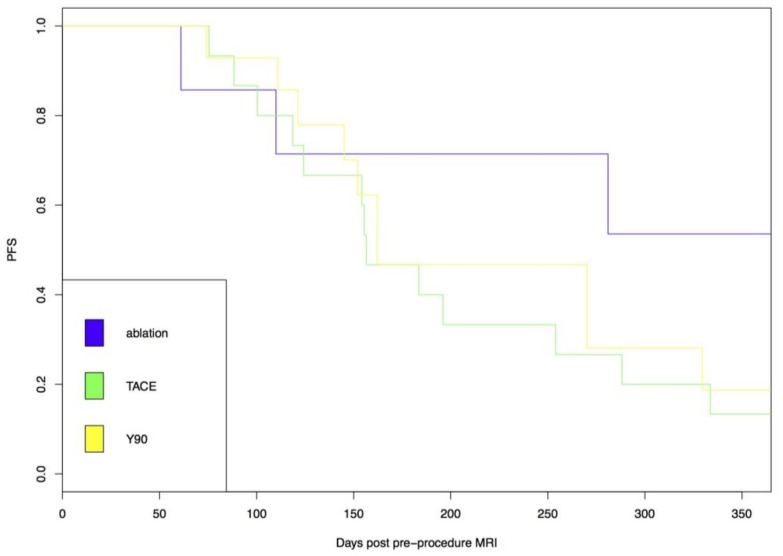
Index lesion specific progression free survival (PFS) was no different following drug eluting embolic (DEE), chemoembolization or radioembolization in low apparent diffusion coefficient (ADC) tumors.

The influence of procedure type on index lesion specific PFS was then evaluated for patients with low ADC values. No significant difference in outcomes was seen between patients with low ADC tumors who underwent DEE chemoembolization or radioembolization (Figure 3).

## 4. Discussion

In this study, DW-MRI was evaluated as an imaging biomarker for OR and index lesion specific PFS in patients with HCC undergoing DEE chemoembolization and radioembolization. ADC values calculated from DW-MRI are inversely correlated with the tumor microenvironmental property of TIP [6]. Elevated TIP is a fundamental barrier to cancer therapy [7,8,9]. TIP is the consequence of several pathologic processes within tumoral tissue, including leaky tumoral blood vessels, absence of lymphatics, and growth-induced stress [9]. By diminishing convective transport gradients and increasing tumoral vascular resistance, TIP diminishes the delivery of cancer therapies to their target.

The concept of ADC maps as a surrogate for TIP provides a biological rationale for this study’s findings that low tumoral ADC is associated with worse immediate OR and PFS. Interestingly, there was no significant difference in PFS by low ADC tumors that underwent either DEE chemoembolization or radioembolization. One might hypothesize that based on their mechanisms of action, radioembolization would be more effective in low ADC tumors compared to DEE chemoembolization. That is, while the delivery of doxorubicin in DEE chemoembolization is substantially affected by TIP [8], radiation emitted by yttrium-90 is not similarly reliant on convention or diffusion to reach its target. However, the increase in vascular resistance caused by TIP would affect therapeutic delivery for both procedures. Moreover, with increased extravasated plasma proteins, proteoglycans, hyaluronans, collagen fibers, and proliferating tumor cells within the tumor interstitium [9], it is possible that the higher tissue density within a low ADC tumor decreases the mean free path gamma rays, narrowing the effective treatment area.

While low ADC was found to correlate with poorer outcomes in this study, this observation has not been seen consistently in previously published reports [12]. Multiple studies have investigated the evaluation of pre-treatment ADC values to accurately predict response to chemoembolization with varying results [13,14,15,16]. Three of these studies have shown that tumors with lower ADC values were more favorable to treatment response than those with higher ADC values [13,14,15]. These authors hypothesize that higher ADC values are representative of tumoral hypoxia and necrosis, two important barriers to cancer therapy [17]. However, the ADC value below which statistically significant overall response was observed varied significantly between these studies. For example, Yuan et al. reported 1.618 × 10^−3^ mm^2^/s [15]. Dong et al. reported a threshold of 1.3 × 10^−3^ mm^2^/s [14]. More recently, Kokabi et al. demonstrated that an ADC value lower than 0.83 × 10^−3^ mm^2^/s predicts OR with high sensitivity (91%) and specificity (96%) [13]. Alternatively, Mannelli et al. demonstrated that tumors with a lower pre- transarterial chemoembolisation (TACE) ADC exhibited poor and incomplete treatment response compared to those with higher pre-TACE ADC values [16]. Other studies found no correlation between pre-treatment ADC values and treatment outcomes [18,19]. Additionally, studies have investigated the utilization of pre-procedural ADC values in patients undergoing radioembolization but have failed to demonstrate the utility of baseline ADC values in predicting tumor necrosis [20]. For patients undergoing ablation, Mori et al. found that patients whose lesions appeared hypointense relative to liver parenchyma on ADC maps to have a significantly higher rate of local recurrence and lower rate of survival [21].

There is a similar lack of clarity on the predictive value of ADC in other cancers. For example, low pre-treatment ADC values correlate with validated tissue and clinical biomarkers for poor prognosis in breast [22] and prostate cancer [23], while higher ADC has been shown to have a better prognosis in rectal cancer [24]. Further confounding the interpretation of ADC values is the variability in ADC measurements. Differences in MRI systems and scanning techniques can lead to substantial differences in ADC values [25]; moreover, ADC maps are often low resolution and noisy, leading to intra- and inter-observer variability [26]. While whole tumor regions of interest are promoted as the most reproducible method of measuring ADC values, this approach does not recognize tumoral heterogeneity. Indeed, identifying discrete regions within tumors with low ADC values may be more effective in predicting areas of treatment resistance (Figure 1).

There are several limitations to this study. Due to the study’s retrospective design, the accuracy of the data is limited by reporting bias. The retrospective nature also prevents control over the time intervals between the procedure and the pre- and post-imaging studies. Our sample size is limited due to the fact that triple phase contrast enhanced CT scanning has been the standard approach for imaging HCC at the study’s institution, excluding numerous patients from this study as they did not undergo pre-procedure MRI. Moreover, diffusion weighted imaging was not standardly performed during liver MRI procedures until approximately 2012, further limiting the study size. For intra-arterial therapies, tumoral vascularity on angiography is an important determinant of treatment response, but due to the retrospective nature of this study, we were unable to randomize patients based on this property.

In conclusion, despite the expanding armamentarium of percutaneous and endovascular therapy options for HCC, predictive tools to identify which patients would benefit from various treatment options are lacking. Diffusion weighted imaging, as a surrogate for tumoral biologic properties such as cell density and TIP, has the potential to identify the presence of intratumoral mechanisms of treatment resistance. In this study, we found that low pre-procedure ADC value is an independent predictor of poorer immediate OR and index lesion specific PFS in patients with HCC undergoing DEE chemoembolization or radioembolization. This knowledge could help guide clinical decision making regarding the most appropriate treatment choice. It could also stimulate explorations into methods to suppress these barriers and improve drug delivery [27].

## Figures and Tables

**Table 1:** Demographics of the study population.

	DEE Chemoembolization	Radioembolization	*P*
Patients (*N*/total, % total)	32/58 (56%)	26/58 (44%)	
Age (median, (range)in yrs)	60 (21–81)	69 (15–80)	0.06
Gender (M/F)	27/5	19/7	0.34
Index lesion size (median, (range) in cm)	4 (1–17)	6.7 (1.7–21.3)	<0.001
Intrahepatic tumor multifocality			0.12
Single tumor	10/32	4/26	
2–3 tumors	10/32	5/26	
>3 tumors	12/32	17/26	
Presence of extra-hepatic disease (*N*/total)	1/32	8/26	0.007
Presence of ascites (*N*/total)	6/32	4/26	1.0
Presence of vascular invasion (*N*/total)	6/32	11/26	0.08
Presence of lymph node metastasis (*N*, % total)	0/32	6/26	0.005
ADC value (median, (range) in 10^−3^ mm^2^/s)	1.08 (0.528–2.71)	0.99 (0.42–2.18)	0.25
ECOG status			0.42
ECOG 0	16/32	11/26	
ECOG 1	14/32	15/26	
ECOG 2	2/32	0/26	
History of hepatic encephalopathy (*N*, % total)	3/32	0/26	0.24
Pre-procedure total bilirubin (median, (range) in mg/dL)	1 (0.3–2.6)	0.75 (0.3–2.5)	0.06
Pre-procedure serum albumin (median, (range) in g/dL)	4.3 (3.0–5.1)	4.2 (3.3–5.3)	0.78
Childs-Pugh score			0.12
Childs-Pugh A	26/32	25/26	
Childs-Pugh B	6/32	1/26	
Childs-Pugh C	0/32	0/26	
BCLC classification			0.02
BCLC A	7/32	0/26	
BCLC B	7/32	5/26	
BCLC C	18/32	21/26	

DEE: drug eluting embolic, ADC: apparent diffusion coefficient, ECOG: extra-hepatic metastases, BCLC: Barcelona Clinic Liver Cancer.

**Table 2:** Univariate and multivariate analysis of demographic and imaging covariates for immediate objective response (OR). On multivariate analysis, apparent diffusion coefficient (ADC) was the only covariate identified that correlated with OR.

	Univariate *P*	Odds Ratio	Multivariate *P*
Age	0.59	-	-
Gender	0.08	2.7 (0.65–11.5)	0.16
Index lesion size	0.86	-	-
Childs-Pugh score	0.61	-	-
Tumor multifocality	0.33	-	-
Presence of vascular invasion	0.53	-	-
ADC ≥ 1.01 × 10^−3^ mm^2^/s	0.03	4.4 (1.2–18.6)	0.03
ECOG status	0.79	-	-
Pre-procedure total bilirubin	0.30	-	-
BCLC classification	0.20	-	-
Treatment modality	0.15		
Doxorubicin dose	0.44	-	-
Yttrium-90 absorbed dose	0.11	−	−

ADC-apparent diffusion coefficient, ECOG-extra-hepatic metastases, BCLC-Barcelona Clinic Liver Cancer.

**Table 3:** Univariate and multivariate analysis of demographic and imaging covariates for index lesion specific progression free survival (PFS). On multivariate analysis, ADC was the only covariate identified that correlated with PFS.

	Univariate *P*	Hazard Ratio	Multivariate *P*
Age	0.55	-	-
Gender	0.89	-	-
Index lesion size	0.96	-	-
Tumor multifocality	0.14	-	-
Presence of extra-hepatic disease	0.05	1.0 (0.2–4.3)	0.98
Presence of ascites	0.05	2.0 (0.5–4.2)	0.08
Presence of vascular invasion	0.93	-	-
Presence of lymph node metastasis	0.006	2.9 (0.3–15.3)	0.2
ADC ≥ 1.01 × 10^−3^ mm^2^/s	0.006	0.44 (0.23–0.84)	0.01
ECOG status	0.81	-	-
Childs-Pugh score	1.0	-	-
BCLC classification	0.59	-	-
Treatment modality	0.47	-	-
Doxorubicin dose	0.46	-	-
Yttrium-90 absorbed dose	0.44	−	−

ADC-apparent diffusion coefficient, ECOG-extra-hepatic metastases, BCLC-Barcelona Clinic Liver Cancer.
